# Unveiling FAM111B: A Pan-Cancer Biomarker for DNA Repair and Immune Infiltration

**DOI:** 10.3390/ijms26073151

**Published:** 2025-03-28

**Authors:** Fang Wei, Wanying Li, Ting Zhou, Xianglin Yuan, Lihong Zhang

**Affiliations:** Department of Oncology, Tongji Hospital, Tongji Medical College, Huazhong University of Science and Technology, Wuhan 430030, China; wfang0223@163.com (F.W.); liwanying0115@163.com (W.L.); zhouting3332006@163.com (T.Z.); yuanxianglin@hust.edu.cn (X.Y.)

**Keywords:** FAM111B, pan-cancer, DNA repair, homologous recombination repair

## Abstract

Recent evidence indicates that FAM111B is significantly involved in the progression of various cancers. Nonetheless, the potential pan-cancer implications of FAM111B have not been systematically investigated. In this study, FAM111B’s expression and oncogenic potential were studied using TCGA and GTEx data via GEPIA2, TIMER2.0, and STRING tools. Pathway enrichment analyses with the GO, KEGG, Reactome, and WikiPathways databases were conducted to explore its role in cancer development. The results were validated via multiplex immunofluorescence assays of pancreatic cancer tissues, microarray assays of ovarian cancer tissues, and protein transcriptomics of ovarian cancer cells. The expression levels of FAM111B were elevated in most cancer types and were associated with poor prognostic outcomes. Mechanistically, FAM111B expression was positively correlated with the expression of genes involved in DNA homologous recombination repair and with the infiltration of Th2 CD4+ T cells. These observations were further substantiated in ovarian cancer cell lines and tissue specimens from pancreatic and ovarian cancers. FAM111B functions as a biomarker for the DNA repair pathway and Th2 CD4+ T-cell infiltration in human malignancies.

## 1. Introduction

The FAM111 trypsin-like peptidase B (FAM111B) gene, located on chromosome 11q12.1, encodes a protein with a trypsin-like peptidase domain for protein hydrolysis [1,2]. It is expressed in multiple human tissues, such as the liver, lung, and pancreas. In 2013, a mutation in FAM111B was linked to hereditary fibrosing poikiloderma with tendon contractures, myopathy, and pulmonary fibrosis (POIKTMP) [1]. Recent evidence demonstrates that FAM111B, which also has been referred to as a cancer-associated nucleoprotein, plays a crucial role in promoting various cancers, such as breast, lung, and pancreatic cancer (PC) [3,4,5,6,7,8,9]. In our previous study, we demonstrated that FAM111B is significantly overexpressed in ovarian cancer (OV) compared to adjacent non-cancerous tissues, and that its expression is linked to an aggressive pathology and poor prognosis [10]. FAM111B has also been shown to promote proliferation, migration, and metastasis in breast, lung, ovarian, and prostate cancer cells, and to accelerate epithelial–mesenchymal transition in breast cancer [3,11,12,13].

Recent research suggests that FAM111B promotes tumorigenesis by modulating DNA damage repair and altering the immune microenvironment. FAM111B has been demonstrated to break down DNA–protein crosslinks, which can interfere with DNA processes [14]. Gene set enrichment analysis (GSEA) further suggested that FAM111B is involved in nucleotide and base excision repair mechanisms in esophageal cancer and PC [13,15]. Additionally, research on lung adenocarcinoma and thyroid cancer has demonstrated that FAM111B is associated with immune-cell infiltration and positively correlates with immune checkpoints, including PDL-1 and CTLA-4 [16,17]. Our previous study of 141 OV cases confirmed the positive correlation between FAM111B and PDL-1 expression [10]. However, the broad application of these findings across cancer types has not been evaluated.

In this study, we conducted a pan-cancer analysis using TCGA data to investigate FAM111B’s role in DNA damage repair and the immune microenvironment across cancers. Our results were validated through immunofluorescence experiments and tissue microarrays of 83 PC and 125 OV samples, as well as protein transcriptomics of OV cells. This study represents the first comprehensive analysis of FAM111B’s role in facilitating DNA damage repair and modulating tumor immunity within a pan-cancer context.

## 2. Results

### 2.1. Elevated Expression of FAM111B Is Correlated with Aggressive Clinicopathological Features and Poor Prognosis in Pan-Cancers

**Figure 1:**
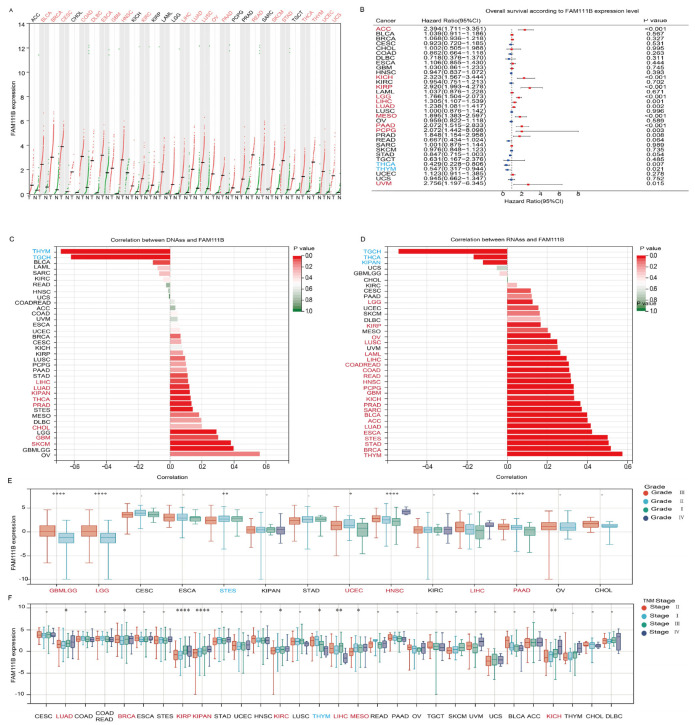
Evaluation of the prognostic value and clinical significance of FAM111B. (**A**) Differential expression of FAM111B between tumor (T) and normal (N) tissue in 31 cancer types. MESO and UVM were excluded due to the absence of corresponding healthy control samples. (**B**) Survival analysis based on FAM111B expression. (**C**) Correlation between FAM111B expression and DNA expression-based stemness score (DNAss). (**D**) Correlation between FAM111B expression and RNA expression-based stemness score (RNAss). (**E**) Association between FAM111B expression and pathological grade. (**F**) Relationship between FAM111B expression and TNM stage. Data were obtained from TCGA and GTEx databases. Red font indicates cancers with elevated FAM111B expression and blue font indicates cancers with reduced FAM111B expression. Statistical significance was established at a threshold of *p* < 0.05, with the following designations: * for *p* < 0.05, ** for *p* < 0.01, and **** for *p* < 0.0001.

To evaluate pan-cancer FAM111B expression, we analyzed RNA-seq data from healthy controls and patients with 31 types of cancer from the TCGA and GTEx datasets. The results revealed that FAM111B mRNA expression is upregulated in 21 tumor types (Figure 1A). Prognostic analysis suggested that elevated FAM111B expression may function as an adverse prognostic biomarker in 10 cancer types, ACC, KICH, KIRP, LGG, LIHC, LUAD, MESO, PAAD, PCPG, and UVM (Figure 1B). Further analysis showed that FAM111B is significantly positively correlated with DNAss in 11 tumor types and RNAss in 26 tumor types, indicating an association with more aggressive tumor behavior (Figure 1C,D). Additionally, FAM111B expression correlated with higher pathological grades in six tumor types (GBMLGG, LGG, UCEC, HNSC, LIHC, PAAD) and higher TNM stages in eight tumor types (LUAD, BRCA, KIRP, KIRPN, KIRC, LIHC, MESO, KICH) (Figure 1E,F). These findings suggest that FAM111B is highly expressed in the majority of tumors, with correlations with aggressive features and poor prognosis in several cancer types.

### 2.2. FAM111B Is Integral to the DNA Replication Process Across Various Cancer Types

**Figure 2:**
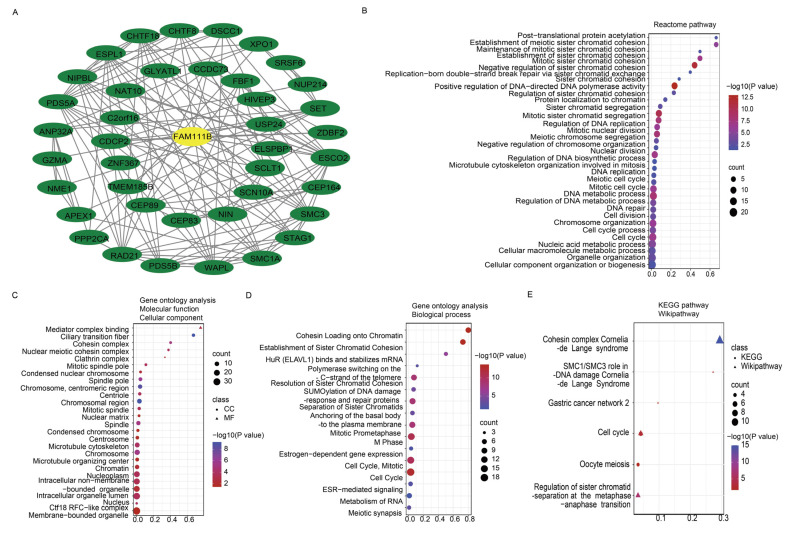
Protein–protein interaction (PPI) network and enrichment analysis pertaining to FAM111B and co-expressed genes. (**A**) PPI network involving FAM111B and 40 associated genes. (**B**–**E**) Enrichment analysis of FAM111B and its related genes, utilizing the Reactome Pathway (panel **B**), Gene Ontology (GO) (panels **C**,**D**), Kyoto Encyclopedia of Genes and Genomes (KEGG), and WikiPathways (panel **E**) databases.

**Figure 3:**
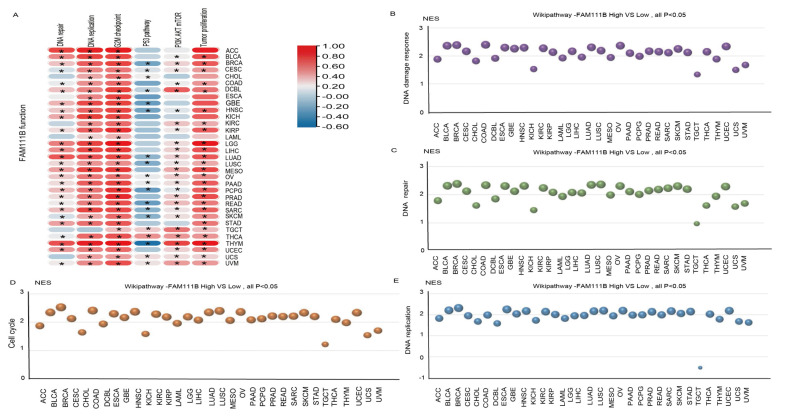
Involvement of FAM111B in the DNA repair pathway. (**A**) The functional pathway of FAM111B according to ssGSEA analysis of TCGA data. (**B**–**E**) Roles of FAM111B in the DNA damage response, DNA repair, cell-cycle regulation, and DNA replication pathways. GSEA analysis of high and low FAM111B expression groups was performed using Wikipathway.

**Figure 4:**
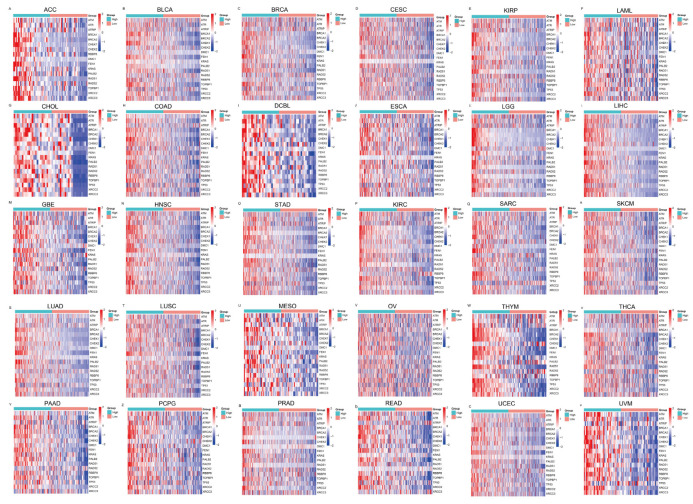
Heatmaps showing the association between FAM111B and homologous recombination repair genes, as derived from the TCGA database. The relationships are depicted across multiple cancer types: (**A**–**F**) ACC, BLCA, BRCA, CESC, KIRP, and LAML; (**G**–**L**) CHOL, COAD, DCBL, ESCA, LGG, and LIHC. (**M**–**R**) GBE, HNSC, STAD, KIRC, SARC, and SKCM. (**S**–**X**) LUAD, LUSC, MESO, OV, THYM, and THCA. (**Y**–**d**) PAAD, PCPG, PRAD, READ, UCEC, and UVM.

To further evaluate the pan-cancer role of FAM111B in the DNA replication process, we constructed a protein–protein interaction (PPI) network graph, which identified interactions between FAM111B and 40 other genes, including genes that encode (SMC3, SMC1A) and regulate (ESCO2) the cohesion complex (Figure 2A) [18], an integral component of the replication fork [19]. Functional enrichment analyses of the 40 FAM111B-interacting genes were conducted using GO, KEGG, Reactome, and WikiPathways. The findings indicate that FAM111B is crucial in genetic information processing, playing roles in DNA replication, DNA repair, sister chromatid segregation, and double-stranded DNA break repair, among others (Figure 2B–E). Using ssGSEA analysis of the TCGA database, we discovered that FAM111B is positively associated with DNA repair, replication, G2M checkpoint, P13K/AKT/mTOR, and tumor proliferation pathways in most cancers, and it is negatively associated with the P53 pathway (Figure 3A). For additional verification, we categorized the tumor samples into FAM111B high- and FAM111B low-expression groups and conducted GSEA analysis using the Wikipathway database. The results confirmed FAM111B’s significant role in DNA repair, the damage response, the cell cycle, and replication across TCGA tumors (Figure 3B–E, all *p* < 0.05). Co-expression heatmaps for homologous recombination-related genes and FAM111B (Figure 4) revealed a positive correlation in 30 of 33 TCGA tumors (all except KIHC, TGCT, and UCS). Therefore, we hypothesize that FAM111B plays a role in the DNA homologous recombination (HR) repair pathway.

### 2.3. FAM111B Plays a Crucial Role in Facilitating DNA Damage Repair Across Various Cancer Types

**Figure 5:**
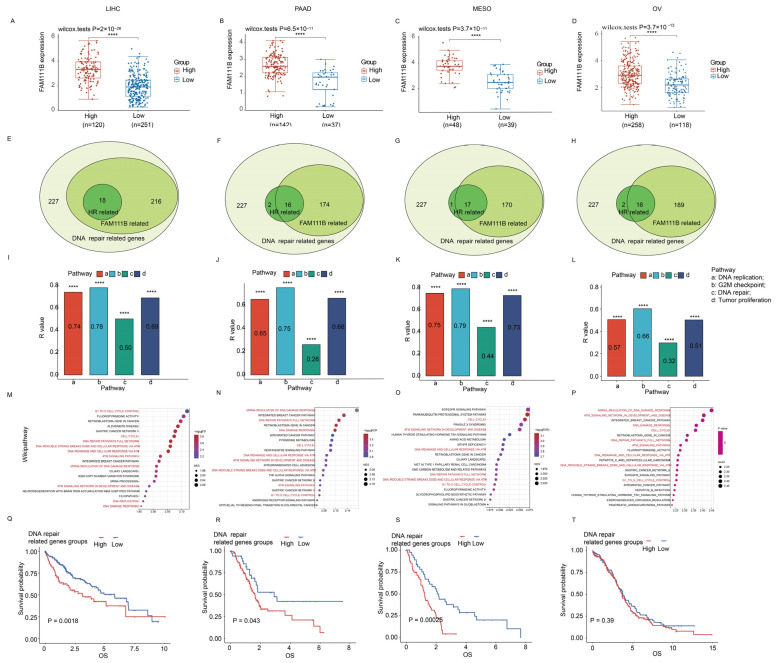
FAM111B’s role in DNA repair across LIHC, PAAD, MESO, and OV. (**A**–**D**) FAM111B expression in high/low DNA repair gene groups. (**E**–**H**) Venn diagram analysis of FAM111B expression among 227 DNA repair–related genes (light green). The numbers of genes with high FAM111B expression (medium green) and those that are homologous recombination (HR) repair genes (dark green) are shown. (**I**–**L**) ssGSEA enrichment of FAM111B expression in four cancer-associated pathways. (**M**–**P**) GSEA enrichment of FAM111B in the Wikipathway database. Pathways associated with DNA damage response are indicated in red. (**G**–**T**) Kaplan–Meier analysis of DNA repair gene expression in LIHC, PAAD, MESO, and OV. Statistical significance was established at a threshold of *p* < 0.05, with the following designations: **** for *p* < 0.0001.

For additional insight into the role of FAM111B in DNA damage repair, we conducted the focused transcriptomic analysis of 227 DNA repair genes in LIHC, PAAD, MESO, and OV patients in the TCGA database. The patients were grouped into high and low DNA repair gene expression clusters (k = 2). Our analysis showed that FAM111B was significantly upregulated in the high-expression cluster compared to the low-expression cluster across all four tumor types (Figure 5A–D; all *p* < 0.05). Furthermore, correlation analyses indicated that 170–216 of the 227 DNA damage repair genes and 16–18 HR-related genes were positively correlated with FAM111B expression (Figure 5E–H). Additionally, ssGSEA analysis suggested that FAM111B positively influences DNA replication, G2M checkpoint control, DNA repair, and tumor proliferation pathways in LIHC, PAAD, MESO, and OV (Figure 5I–L, all *p* < 0.05), while GSEA analysis revealed FAM111B’s active role in DNA damage response pathways, including G1 cell-cycle control, the complete DNA repair network, DNA double-strand breaks, and ATR-mediated responses (Figure 5M–P; all *p* < 0.05). For additional prognostic insight, we performed a survival analysis of the high and low DNA repair gene expression groups. The results showed significantly shorter overall survival times in the high-expression groups for LIHC, PAAD, and MESO (*p* < 0.05), but not for OV (*p* > 0.05) (Figure 5Q–T). These findings indicate that FAM111B is closely linked to and regulates genes involved in DNA damage repair.

### 2.4. Multiplex Immunofluorescence Analysis Demonstrates That Pan-Cancer FAM111B Expression Is Positively Correlated with DNA Damage Repair Pathways and BRCA1 Expression

**Figure 6:**
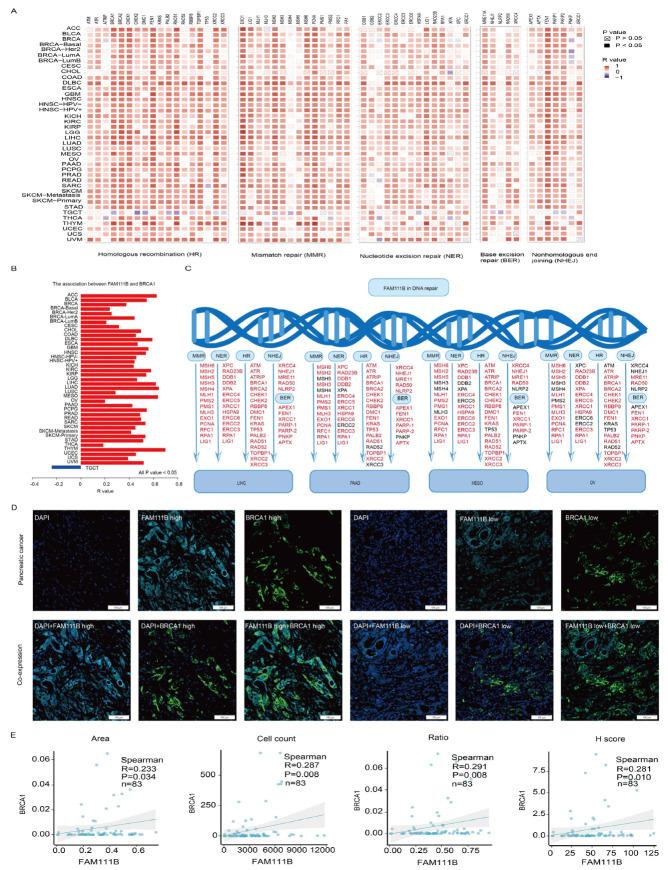
The positive correlation between FAM111B and genes involved in DNA repair. (**A**) The positive correlation between FAM111B and sets of genes associated with specific DNA damage processes. Correlations were evaluated according to tumor type using data in TCGA and GTEx databases. (**B**) The correlation between FAM111B and BRCA1 across various cancer types. (**C**) The correlation between FAM111B and DNA repair genes specifically within LIHC, PAAD, MESO, and OV. (**D**) Representative images of FAM111B and BRCA1 immunofluorescence staining in PC and adjacent tissues. (**E**) The correlation between FAM111B and BRCA1 across the area (image section), cell count (number of positive cells), H-score (immunofluorescence assessment), and ratio (number of positive cells divided by the total cells). The H-score was calculated as ((intensity of 1 + cell positive rate) × 1 + (intensity of 2 + cell positive rate) × 2 + (intensity of 3 + cell positive rate) × 3) × 100.

To further clarify FAM111B’s role in DNA damage repair, we examined its relationship with key proteins involved in various DNA repair mechanisms, including HR, mismatch repair (MMR), nucleotide excision repair (NER), base excision repair (BER), and nonhomologous end joining (NHEJ), which demonstrated positive correlations across multiple cancers (Figure 6A). Moreover, FAM111B and BRCA1 were positively correlated in 39 tumors; the exception was a negative correlation in TGCT (Figure 6B, all *p* < 0.05). In LIHC, PAAD, MESO, and OV, FAM111B also was positively correlated with HR, MMR, NER, BER, and NHEJ repair proteins (Figure 6C). To confirm the correlation between FAM111B and BRCA1, we performed a multiplex immunofluorescence analysis of 83 PC samples (Figure 6D). The results verify positive correlation between FAM111B and BRCA1 across the area, cell count, ratio, and H-score metrics (Figure 6E, all *p* < 0.05), further supporting the role of FAM111B in HR.

### 2.5. FAM111B Knockdown Modulates DNA Repair Processes

**Figure 7:**
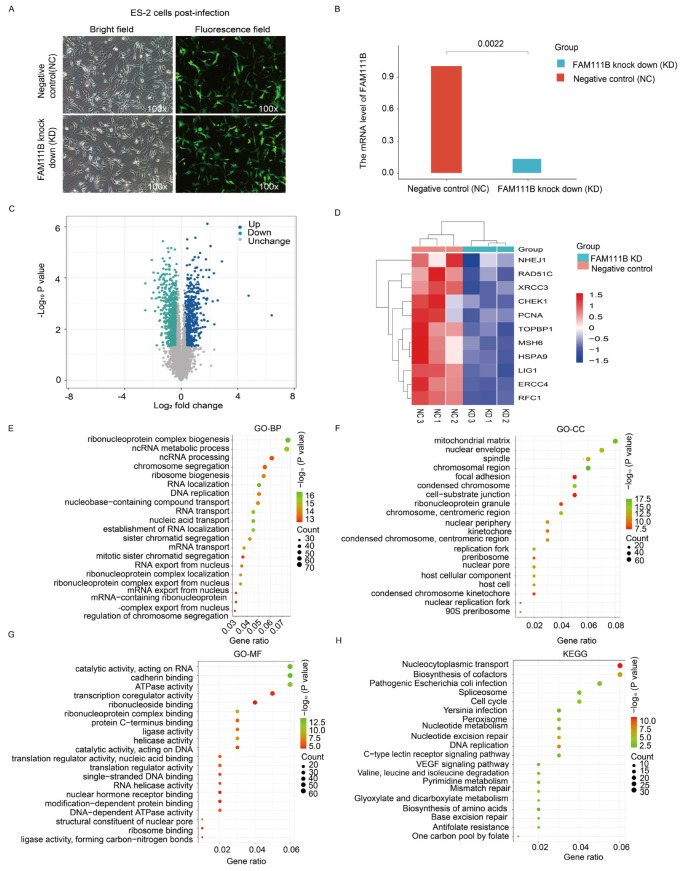
Transcriptomic analysis of FAM111B knockdown cells. (**A**) Fluorescence image of ES-2 cells infected with a negative control siRNA (NC) or FAM111B knockdown siRNA (KD). (**B**) qPCR detection of FAM111B levels in infected cells. (**C**) Volcano plot of gene expression changes in NC versus KD cells. (**D**) Heat map of differentially expressed DNA repair genes. (**E**–**H**) Enrichment analysis of differentially expressed genes in GO (BP, biological processes; CC, cellular component; MF, molecular function) and KEGG databases.

To verify the role of FAM111B in DNA repair pathways, we transfected ES-2 ovarian cancer cells with FAM111B siRNA lentivirus to silence FAM111B expression (Figure 7A). The qPCR results indicated an 86.63% reduction in FAM111B expression in the knockdown (KD) group compared to the negative control group (NC) (Figure 7B). Furthermore, a volcano plot analysis revealed 535 up-regulated and 864 down-regulated genes between the FAM111B-KD and NC cells (Figure 7C). Notably, heat maps showed a reduced expression of HR-related genes, including NHEJ1, RAD51C, and XRCC3, in the FAM111B-KD group (Figure 7D; all *p* < 0.05). Enrichment analysis of the 864 down-regulated genes, using the GO database, confirmed that FAM111B knockdown modulates genetic information processing activities, such as ncRNA metabolic processes and chromosome segregation (Figure 7E–G). Furthermore, KEGG analysis indicated that FAM111B knockdown modulates DNA repair mechanisms, including MMR and BER (Figure 7H). Therefore, our data confirm the close association of FAM111B with DNA damage repair in OV.

### 2.6. FAM111B Modulates the Anti-Tumor Immune Microenvironment Across Various Cancer Types

**Figure 8:**
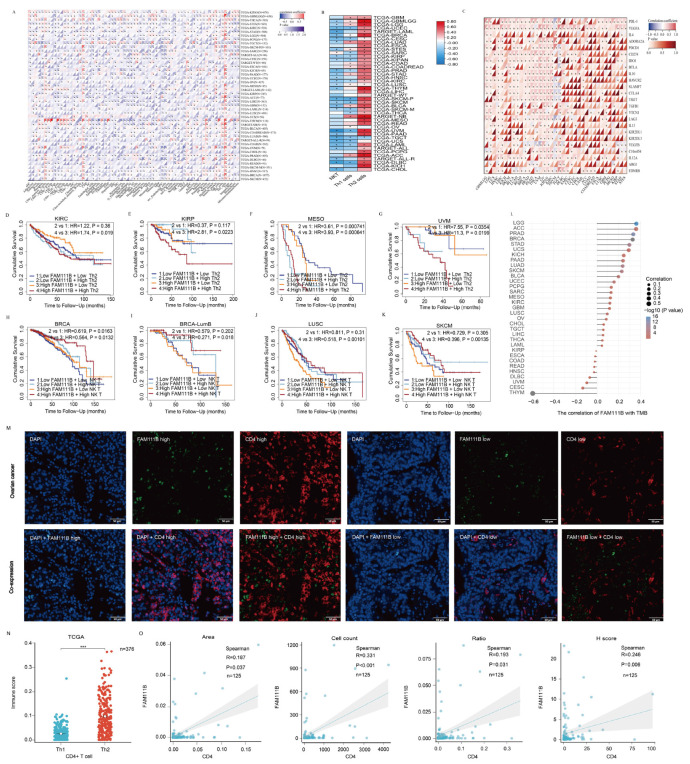
The role of FAM111B in the tumor immune microenvironment. (**A**) FAM111B’s association with immune cell marker expression in different types of cancer. (**B**) Correlation between FAM111B expression and NK T, Th1, and Th2 cell marker enrichment. (**C**) Correlation between FAM111B expression and immune checkpoint markers. (**D**–**G**) The prognostic impact of FAM111B and Th2 cells in specific cancer types was evaluated according to Kaplan-Meier survival analysis of high/low FAM111B and CD4 expression groups. (**H**–**K**) The prognostic impact of FAM111B and NK T cells in specific cancer types was evaluated according to Kaplan-Meier survival analysis of high/low FAM111B and NK marker expression groups. (**L**) The association between FAM111B expression and the tumor mutation burden (TMB). (**M**) Representative immunofluorescence staining of FAM111B and CD4 expression for high- and low-expression samples. (**N**) Th1 and Th2 CD4+ T−cell infiltration scores for OV samples from the TCGA database. (**O**) FAM111B correlation with CD4 in OV samples according to the area, cell count, H-score, and ratio. *** for *p* < 0.001.

To investigate FAM111B’s role in the tumor immune microenvironment, we examined its correlation with the infiltration of 64 immune cell types using TCGA data (Figure 8A). FAM111B was negatively correlated with NK T cells and Th1 CD4+ T lymphocytes but positively correlated with Th2 CD4+ T lymphocytes in most tumors (Figure 8B). In addition, FAM111B showed a positive correlation with PDL-1, CTLA-4, and other immunosuppressive checkpoints in most tumors (Figure 8C). We evaluated the prognostic value of Th2 and NK T cells alongside FAM111B expression, which indicated that Th2 enrichment predicted a worse prognosis in KIRC, KIRP, MESO, and UVM, while a reduced number of NK T cells indicated a worse prognosis in BRCA, LUSC, and SKCM, particularly in the high FAM111B expression group (Figure 8D–K). Finally, we investigated the association between FAM111B expression and the tumor mutation burden (TMB). The results identify a positive correlation with LGG, ACC, PRAD, and several other tumor types (Figure 8L). Consequently, we hypothesize that elevated FAM111B expression may modulate the anti-tumor immune microenvironment, thereby facilitating tumor malignancy progression. To further explore the relationship between FAM111B and the anti-tumor immune microenvironment, we conducted multiplex immunofluorescence experiments using tissue microarrays from 125 OV patients (Figure 8M). FAM111B was positively correlated with CD4+ T cells in terms of the area, cell count, ratio, and H-score (Figure 8N, *p* < 0.05). Moreover, the analysis of 376 OV cases in the TCGA database suggested that Th2 CD4+ T cells were found to be significantly more prevalent than Th1 CD4+ T cells in OV, indicating a Th2 dominance among CD4+ T cells (Figure 8O, *p* < 0.05). These findings suggest that FAM111B may exert a chemotactic effect on CD4+ T cells in OV, with a possible preference for Th2 CD4+ T cells.

## 3. Discussion

In our pan-cancer analysis, we demonstrated that FAM111B is linked to malignancy and poor prognosis, and that alterations in DNA repair and the immune microenvironment underlie the severity of diseases. FAM111B’s role in DNA repair was verified in LIHC, PAAD, MESO, and OV. Multiplex immunofluorescence assays showed a positive correlation between FAM111B and the DNA repair protein BRCA1 in PC tissues, while transcriptomic analysis verified FAM111B’s role in DNA repair in OV cells. Furthermore, immunoassays revealed FAM111B’s negative correlation with Th1 CD4+ T cells and positive correlation with Th2 CD4+ T cells, which was verified by multiplex immunofluorescence assays of OV tissues. Thus, our results suggest that FAM111B aids DNA repair and influences Th1/Th2 differentiation in CD4+ T cells, potentially contributing to cancer development across various types.

Evidence has increasingly supported the involvement of FAM111B in the damage response and repair of DNA. For example, proteomic transcriptome analysis demonstrated FAM111B’s role in DNA repair pathways in ESCA cells [15]. Furthermore, GSEA analysis demonstrated that FAM111B is significantly enriched in NER and BER in PC [13]. However, this study is the first to evaluate the role of FAM111B in mediating DNA repair mechanisms across cancers. We evaluated its expression patterns in 33 cancer types and comprehensively assessed the FAM111B-associated pathways, which suggested mechanisms that are generalizable to many different cancer types. Notably, our findings revealed a negative association between FAM111B expression and the P53 pathway. The P53 signaling pathway, when phosphorylated and activated, triggers stress responses like DNA damage [20]. Research shows that the expression of FAM111B is increased during the DNA damage response and is linked to the activated P53 pathway in LUAD [11]. In OV, FAM111B correlates positively with phosphorylated AKT protein, which negatively influenced the P53 pathway [12]. FAM111B has also been reported to interact with CAPNS1, which indirectly regulates FANCD2, a key DNA damage response protein [21,22]. Studies suggest that FAM111B, like its homolog FAM111A, aids in removing DNA protein crosslinks during replication, enhancing DNA repair [2]. Therefore, these results support our findings suggesting that FAM111B influences the DNA damage and repair in multiple types of cancer.

Our research indicates that FAM111B is positively associated with DNA damage repair genes and plays a significant role in the occurrence of poor prognosis. Previous studies have shown that, in breast cancer, the analysis of eight DNA repair-related genes, including RPA3 and XRCC4, among others, reveals that patients exhibiting high expression levels of these genes tend to have poorer prognoses [23]. Similarly, elevated Rad51 expression is correlated with adverse outcomes in OV [24]. Active DNA damage repair mechanisms enable cancer cells to survive under conditions of DNA damage and may introduce mutations during the repair process, potentially activating oncogenes or inactivating tumor suppressor genes and thereby accelerating tumor progression [25,26]. Moreover, the accumulation of such mutations provides tumor cells with a growth advantage, driving their clonal evolution and enhancing their invasive and metastatic potential [27].

The targeting of key proteins, such as PARP, in DNA damage repair has been demonstrated to cause “synthetic lethality” by exploiting HR deficiencies, leading to tumor cell death [28]. Furthermore, mutations in HR-related genes, such as BRCA1/2, have been demonstrated to cause HR deficiency, thus increasing the effectiveness of PARP inhibitors [29]. In this study, we demonstrated that FAM111B expression correlates with 17 HR repair proteins, including ATM, ATR, and BRCA1/2, in 30 out of 33 of the studied cancers. The positive correlation between FAM111B and BRCA1 was verified by multiplex immunofluorescence assays of 83 PC samples. Based on these findings, the inhibition of FAM111B may induce HR deficiency and increase PARP inhibitor sensitivity. Research indicates that the knockout of FAM111A, a structurally and functionally similar protein, boosts PARP inhibitor effectiveness [30]. Given that FAM111B shares a 43% sequence similarity and structural domains with FAM111A, it may perform a similar role [1,2,31]. Moreover, our research showed that FAM111B deficiency may lead to HR deficiency by targeting multiple DNA damage and repair pathways. Therefore, the inhibition of FAM111B in patients with high FAM111B expression could potentially provide an approach for improving their treatment by disrupting the DNA damage response. FAM111B inhibitors are not yet available for clinical examination; however, the HERB database predicts that 12 natural products, including oleum anisistellati, fructus cinnamomi cassiae, and cassia bark oil, are potential FAM111B inhibitors [32]. Therefore, our findings could provide insights for the development of natural remedies for cancer treatment.

Our research also demonstrated that FAM111B is negatively correlated with Th1 CD4+ T cells and positively correlated with Th2 CD4+ T cells across various cancers, indicating its role in shifting the balance of these cell populations. The functions of Th1 and Th2 populations are distinctly different, with Th1 cells boosting anti-tumor immunity and Th2 cells aiding in tumor immune evasion [33], although both cell types originate from CD4+ T cells and inhibit each other to maintain immune system balance [34]. In the tumor microenvironment, a shift from Th1 to Th2 immune responses has been shown to influence cancer progression [35]; increased Th2 and decreased Th1 levels are linked to poor prognosis [36,37]. Moreover, studies have shown that FAM111B affects the T-cell balance in thyroid cancer and LUAD [16,17]. Therefore, our findings are consistent with the established role of FAM111B in modulating the immune environment to promote cancer progression.

Our study is constrained by several limitations. In the experimental validation phase, we exclusively utilized tissue microarrays of PC and OV cells to investigate the role of FAM111B in DNA repair. However, further validation across a broader range of cancer types is warranted. Additionally, within the tumor microenvironment, we observed a correlation between FAM111B and CD4+ T cells in the OV tissue microarrays; however, our analysis did not distinguish between specific subtypes, such as Th1 and Th2 cells. This limitation will be addressed in future research efforts.

## 4. Materials and Methods

### 4.1. Patient Cohorts

In this study, pan-cancer analysis data were sourced from the comprehensive data on various cancer types available in The Cancer Genome Atlas (TCGA) database, which includes 33 distinct cancer types and encompasses a total of 10,228 patients. Based on the TCGA database, BRCA can be subdivided into four subtypes: BRCA-Basal, BRCA-Her2, BRCA-LumA, and BRCA-LumB; HNSC can be divided into two subtypes: HNSC-HPV (−) and HNSC-HPV (+); and SKCM can be divided into two subtypes: SKCM-Metastasis and SKCM-Primary. Therefore, the 33 types of tumors in the TCGA database can be further divided into 41 subtypes, which were used to conduct a correlation analysis between FAM111B and DNA repair proteins or tumor stemness scores. For expression controls, the Genotype-Tissue Expression (GTEx) database (https://www.gtexportal.org/home/) (accessed on 21 September 2023), containing genomic information from 838 healthy donors, was utilized. Furthermore, for comprehensive immune analysis, we expanded our analysis to the Therapeutically Applicable Research to Generate Effective Treatments (TARGET) database (https://ocg.cancer.gov/programs/target) (accessed on 21 September 2023) to incorporate four additional cancer types: osteosarcoma, acute lymphoblastic leukemia, neuroblastoma, and high-risk Wilms tumor.

### 4.2. Differential Expression and Prognostic Evaluation of FAM111B in Pan-Cancers

To analyze FAM111B expression and prognosis, transcription data from TCGA and GTEx were downloaded. Tumor versus normal tissue expression differences were evaluated with ANOVA using GEPIA2. FAM111B’s prognostic value was assessed using a Cox proportional hazards model via TIMER2.0. We retrieved the TCGA pan-cancer tumor stemness scores from the UCSC (https://xenabrowser.net/) (accessed on 21 September 2023) database, excluding cancer types with fewer than three samples, and ultimately acquired expression data for 37 cancer types to explore FAM111B’s link with tumor stemness. The DNA expression-based stemness score (DNAss) and RNA expression-based stemness score (RNAss) were derived for Pearson analysis using previous methylation signatures [18]. FAM111B expression across various pathological grades and TNM stages was analyzed using R software (v3.6.4) with unpaired Student’s *t*-test or ANOVA.

### 4.3. Functional Enrichment Analysis of FAM111B in Various Cancers

Using the STRING online tool, a PPI network was created for FAM111B and 40 related genes. These genes were analyzed using GO, KEGG, Reactome, and Wikipathway. FAM111B-related pathways were further examined using ssGSEA and the “GSVA” package in R, and analyses were based on TCGA data. GSEA with Wikipathway analysis was conducted to validate FAM111B’s pathways across 33 tumors. Heatmaps from an online platform (https://www.bioinformatics.com.cn) (accessed on 15 August 2023) were generated to visualize co-expression patterns of homologous recombination-related genes in groups with high (50–100%) and low (0–50%) FAM111B expression.

### 4.4. Correlation Analysis of FAM111B Within the DNA Repair Pathway in Four Types of Cancers

The “ConsensusClusterPlus” R package was used to analyze 227 DNA damage repair genes in LIHC, PAAD, MESO, and OV, and patients were categorized into high- and low-expression groups. Kaplan–Meier survival and differential expression analyses were performed between these groups, and Spearman analysis was employed to evaluate the correlation between FAM111B and the 227 genes. GSEA and ssGSEA were employed to investigate FAM111B’s functional pathways, while Spearman correlation analysis with TCGA DNA repair proteins was used to examine its role in DNA repair through TIMER2.0.

### 4.5. Immunoassay Investigation of the Role of FAM111B in the Immune Microenvironment

Gene expression profiles for each cancer type were extracted from the TCGA dataset, and immune cell infiltration scores were calculated using the IOBR R package with the xCell algorithm [38]. The prognostic significance of FAM111B’s correlation with Th2 and NK T-cell infiltration was analyzed using TIMER 2.0 and KM mapper (https://kmplot.com/analysis/) (accessed on 18 October 2023). Correlation data between FAM111B and the tumor mutation burden (TMB) were derived using TCGA transcriptome data.

### 4.6. Tissue Microarrays

Tissue microarrays from Shanghai Outdo Biotechnology Co. Ltd. included samples from 83 PC and 125 OV patients, and all clinical samples were clustered with patients’ informed consent. The PC microarrays (HPanA180Su10) included 78 pancreatic ductal adenocarcinoma cases and 5 cases of other subtypes, including adeno-squamous carcinoma, ampullary adenocarcinoma, high-grade intraductal papillary mucinous neoplasm with pancreatic ductal adenocarcinoma, and adenocarcinoma. The OV tissue microarrays (panel HOvaC151Su01) comprised 65 serous adenocarcinoma cases, 30 mucinous adenocarcinoma cases, 13 endometrioid adenocarcinoma cases, and 17 cases of various other subtypes, including clear cell carcinoma, serous-mucinous adenocarcinoma, dysgerminoma, squamous cell carcinoma, borderline mucinous tumors, adenocarcinoma, malignant Brenner tumors, and yolk sac tumors.

### 4.7. Multiplex Immunohistochemistry

Tissue samples were dewaxed in xylene, rehydrated with 10% formalin, and subjected to antigen retrieval in a microwave. Antibodies against FAM111B (Novus, NBP1-86645, Chesterfield, Missouri, USA), BRCA1 (Novus, NB100-404, Missouri, USA), or CD4 (ZSGB, ZM0418, Beijing, China) were applied, and fluorescent signals were stained and amplified using the PANO 7-plex IHC kit (Panovue, Beijing, China). After staining, DAPI solution was applied, and the slides were sealed with mounting medium. Section scanning was conducted using the Olympus VS200 MTL (Olympus, Hamburg, Germany) and UPLXAPO20X lenses. QuPath v0.3.0 software was used for analyzing multi-color fluorescence images.

### 4.8. Cell Culture and FAM111B Gene Knockdown

The ES-2 ovarian cancer cell line was obtained from the American Type Culture Collection (ATCC, Manassas, VA, USA) and cultured in McCoy’s 5A medium with 10% FBS (Ausbian, Cat. No. A11-102) at 37 °C in 5% CO_2_. At 80% confluence, a 10^5^ cells/mL suspension of FAM111B-targeting shRNA lentivirus (sense sequence: 5′-GCCTGCCTAGTGATTCTCATT-3′, anti-sense sequence: 5′-AATGAGAATCACTAGGCAGGC-3′) was applied. As a negative control, a nonsense shRNA lentivirus (target sequence 5′-TTCTCCGAACGTGTCACGT-3′) was applied. After 72 h, the expressed on the reporter gene was assessed by fluorescence microscopy to evaluate infection levels. An infection efficiency of 80% or higher was deemed satisfactory.

### 4.9. Real-Time Quantitative PCR

After centrifugation of cultured cells, the supernatants were removed, and 1 mL of Trizol (Promega, 3111-100, Fitchburg, Wisconsin, USA) per sample was added for cell lysis and RNA extraction. The cDNA was synthesized using the M-MLV Kit (Promega, M1705, Wisconsin, USA), and PCR was performed with SYBR Master Mix. RNA levels were measured using the 2^−ΔΔCT^ method. Primers from Guangzhou Ribobio Co., Ltd. (Guangzhou, China) included GAPDH forward (5′-TGACTTCAACAGCGACACCCA-3′) and reverse (5′-CACCCTGTTGCTGTAGCCAAA-3′); and FAM111B forward (5′-GAGTTCTGCCCTACTCCTGAC-3′) and reverse (5′-AAATCTGTCGCCATAGTCCTG-3′).

### 4.10. Protein Extraction and Transcriptomic Analysis

Proteins were extracted from cells using the SDT lysis method and then quantified using the BCA Protein Assay Kit (P0012, Beyotime, Shanghai, China). UA buffer (8 M Urea, 150 mM Tris-HCl pH 8.5) was added for peptide quantification using the FASP enzymatic digestion method. Mass spectrometry analysis was conducted on the Orbitrap Exploris 480 in positive ion mode, with a parent ion scan range of 350–1200 m/z and a first-level resolution of 120,000. The AGC target was set at 300%, with a first-level IT cap of 50 ms. Mass spectrometry data were collected, processed, and analyzed using data-dependent acquisition and the UniProt protein sequence database (http://www.uniprot.org) (accessed on 15 February 2023). The “enhanced volcano” R package was used to create a volcano plot to visualize gene expression differences between the FAM111B KD and NC groups. The downregulated genes of the FAM111B KD and NC groups were input into the GO and KEGG databases for functional enrichment analysis.

### 4.11. Statistical Analysis

Statistical analysis was performed using R v4.0.3 and ggplot2 v3.3.3 for graphing. Correlations were assessed with Spearman and Pearson analyses, and group differences were evaluated using the unpaired Student’s *t*-test or ANOVA. Statistical significance was established at a threshold of *p* < 0.05, with the following designations: * for *p* < 0.05, ** for *p* < 0.01, *** for *p* < 0.001, and **** for *p* < 0.0001.

## 5. Conclusions

In summary, this study represents the first demonstration that FAM111B facilitates cancer progression by modulating DNA repair processes, particularly HR repair in various cancers. This mechanism has potential therapeutic implications for augmenting the effectiveness of PARP inhibitors through “synthetic lethality”. Furthermore, we demonstrated that FAM111B promotes the transition from Th1 to Th2 cells, thereby suggesting a mechanism for contributing to tumor immune evasion. These findings provide new insights into the oncogenic mechanisms associated with FAM111B expression.

## Data Availability

The public datasets utilized in this study are accessible through online database. Publicly available data were obtained from the TCGA websites (https://portal.gdc.cancer.gov/ or https://xenabrowser.net/datapages/) (accessed on 21 September 2023), GTEx website (https://www.gtexportal.org/home/) (accessed on 21 September 2023), and TARGET database (https://ocg.cancer.gov/programs/target) (accessed on 21 September 2023).

## Abbreviations

FAM111B	the FAM111 trypsin like peptidase B
POIKTMP	hereditary fibrosing poikiloderma with tendon contractures, myopathy, and pulmonary fibrosis;
PC	pancreatic cancer
OV	ovarian cancer
DPC	DNA-protein crosslinking complexes
TCGA	the cancer genome atlas database
mIF	multiple immunofluorescence
GTEx	the genotype-tissue expression
DNAss	DNA expression-based stemness scores
RNAss	RNA expression-based stemness scores
TMB	tumor mutation burden;
HR	homologous recombination
MMR	mismatch repair;
NER	nucleotide excision repair;
BER	base excision repair;
NHEJ	non-homologous end joining repair;
PPI	protein-protein interaction;
GO	gene ontology;
KEGG	kyoto encyclopedia of genes and genomes;
GSEA	gene set enrichment;
CAPNS1	calpain small subunit 1;
DDR	DNA damage repair;
HRD	HR deficiency;
TME	tumor microenvironment;
LAML	acute myeloid leukemia;
ACC	adrenocortical carcinoma;
BLCA	bladder urothelial carcinoma;
LGG	brain lower grade glioma;
BRCA	breast invasive carcinoma;
CESC	cervical squamous cell carcinoma and endocervical adenocarcinoma;
CHOL	cholangiocarcinoma;
COAD	colon adenocarcinoma;
GBM	glioblastoma multiforme;
HNSC	head and neck squamous cell carcinoma;
KICH	kidney chromophobe;
KIRC	kidney renal clear cell carcinoma;
KIRP	kidney renal papillary cell carcinoma;
LIHC	liver hepatocellular carcinoma;
LUAD	lung adenocarcinoma;
LUSC	lung squamous cell carcinoma;
DLBC	lymphoid neoplasm diffuse large B-cell lymphoma;
MESO	mesothelioma;
PAAD	pancreatic adenocarcinoma;
PCPG	pheochromocytoma and paraganglioma;
PRAD	prostate adenocarcinoma;
READ	rectum adenocarcinoma;
SARC	sarcoma
SKCM	skin cutaneous melanoma
STAD	stomach adenocarcinoma
TGCT	testicular germ cell tumors;
THYM	thymoma;
THCA	thyroid carcinoma;
UCS	uterine carcinosarcoma;
UCEC	uterine corpus endometrial carcinoma;
UVM	uveal melanoma.
